# In vivo imaging of the spatial heterogeneity of intratumoral acidosis (pH) as a marker of the metastatic phenotype in breast cancer

**DOI:** 10.1186/s13058-025-02065-y

**Published:** 2025-06-23

**Authors:** Alessia Corrado, Nicla Lorito, Annasofia Anemone, Antonella Carella, Daisy Villano, Elisa Pirotta, Francesco Gammaraccio, Angela Subbiani, Marina Bacci, Walter Dastrù, Andrea Morandi, Dario Livio Longo

**Affiliations:** 1 Institute of Biostructures and Bioimaging (IBB), National Research Council of Italy (CNR), Via Nizza 52, Turin, 10126 Italy; 2 Department of Experimental and Clinical Biomedical Sciences, University of Florence, Viale Morgagni 50, Florence, 50134 Italy; 3 Department of Molecular Biotechnology and Health Sciences, University of Turin, Via Nizza 52, Turin, 10126 Italy

**Keywords:** Tumor metabolism, Tumor acidosis, Breast cancer, Imaging, MRI, Chemical exchange saturation transfer (CEST), Metastatic potential

## Abstract

**Background:**

Metabolic alterations, including acidosis in the tumor microenvironment, have been extensively linked to more aggressive phenotypes and increased therapy resistance. However, current imaging techniques are limited in their ability to capture extracellular tumor acidosis precisely and assess spatial heterogeneity in vivo, making its association with augmented malignancy poorly understood. In this study, we investigated whether Magnetic Resonance Imaging– Chemical Exchange Saturation Transfer (MRI-CEST) technique for tumor pH imaging of intratumoral acidosis could differentiate between metastatic and non-metastatic breast cancers.

**Methods:**

Isogenic metastatic (4T1) and non-metastatic (67NR) breast cancer cell lines were characterized for their metabolic and acidosis features, including LDH-A/PDK-1 expression, glucose consumption, extracellular acidification rate (ECAR) and oxygen consumption rate (OCR). Potential relationship between tumor acidosis, vascularization and hypoxia with metastatic potential was assessed in vivo by MRI-based imaging approaches in orthotopic breast tumors. Validation of MRI findings was assessed ex vivo by western blot, immunohistochemistry and immunofluorescence assays for a multiparametric characterization of tumor microenvironment and metabolic properties.

**Results:**

We observed a higher energetic profile of the 4T1 cells compared to the 67NR cells, alongside elevated glycolytic (LDH-A, PDK-1), hypoxia (CAIX, Pimonidazole), and vascularization (CD31) markers in 4T1 orthotopic primary tumors, which were associated with a greater metastatic propensity. MRI-CEST tumor pH imaging revealed increased extracellular tumor acidity in 4T1 tumors, along with marked spatial intratumoral heterogeneity, in contrast to the more homogenous 67NR tumors, as further confirmed by LAMP-2 staining. Notably, this spatial intratumor heterogeneity in acidosis enables clear differentiation between high- and low-malignancy tumors.

**Conclusions:**

These findings underscore the role of tumor acidosis and its spatial heterogeneity in promoting aggressive phenotypes and highlight the potential of in vivo tumor pH imaging as a marker of malignancy in breast cancers.

**Supplementary Information:**

The online version contains supplementary material available at 10.1186/s13058-025-02065-y.

## Background

Breast cancer is the first malignancy for incidence and cause of cancer-related deaths in women worldwide [1]. The development of breast cancer is characterized by a multistep progression that is strongly influenced by tumor metabolic reprogramming [2]. During cancer development, local hypoxia induces cancer cells to shift towards enhanced glycolysis and increased expression of proton transporters. This results in the accumulation of lactate and H^+^ in the extracellular space, leading to tumor acidosis or low extracellular pH (pH_e_) [3]. Tumor acidosis has been associated with higher invasion and aggressive behavior, since the adaptation to low pH_e_ can select cancer subclones characterized by more malignant phenotypes, with increased proliferation, metastatic ability, and resistance to chemo-, radio-, and immuno-therapies [4, 5].

However, the key role of tumor acidosis in promoting the metastatic behavior of solid tumors has been investigated mainly by *in vitro* andex vivo studies, or by exploiting in vivo imaging approaches that have been proven difficult to translate into the clinic. For example, histologic examinations have shown that tumor borders are acidic and display hallmarks of aggressiveness and invasion [6]. Moreover, optical imaging studies based on window chambers have demonstrated that an acidic pH_e_ is correlated with altered tumor metabolism and invasion [7, 8]. Considering the importance of non-invasive imaging methods for assessing tumor acidosis in living beings and the urgent need to translate these methods to clinical practice, several in vivo methods have been proposed for measuring intra- and extra-cellular tumor pH values [9]. Recently, the Magnetic Resonance Imaging (MRI) - Chemical Exchange Saturation Transfer (CEST) technique, which involves the administration of a pH-responsive contrast agent, has been successfully exploited for measuring in vivo tumor pH_e_ with high accuracy [10]. Several studies exploiting this approach have highlighted in vivo the associations among tumor acidosis, dysregulated glycolysis, and metastatic potential, demonstrating that tumor acidosis is correlated with increased glucose uptake [11] and highly invasive and metastatic behavior in breast cancer mouse models [12]. Furthermore, MRI-CEST tumor pH imaging can be exploited as a novel imaging-based biomarker for monitoring the therapeutic response to novel anticancer therapies [13–19].

Tumors exhibit substantial heterogeneity with spatial variations at several levels (e.g., gene expression, metabolites, proteins, and morphological structure) with profound clinical implications, usually associated with reduced long-term survival [20, 21]. This heterogeneity leads to intratumoral differences in metabolic pathways, vascularization, hypoxia, and acidosis [22, 23]. Furthermore, owing to technical limitations, spatial tumor pH distribution is poorly understood, since many studies have measured bulk pH only or have been limited to small regions / central portions of the tumor [24, 25]. Here, we exploit MRI-CEST tumor pH imaging to assess tumor pH_e_ with high spatial resolution and to monitor spatial heterogeneity in vivo. This approach aims to understand how heterogeneity in intratumoral acidosis correlates with an invasive phenotype.

To this end, we analyzed the metabolic characteristics of two closely related cell lines derived from the same spontaneous mammary adenocarcinoma growing in a BALB/c mouse. These cell lines display different metastatic capabilities: 67NR cells do not metastasize whereas 4T1 cells are overtly metastatic [26].

Previous studies have demonstrated marked differences in lactate production, oxidative phosphorylation, and metabolic plasticity between these two breast cancer cell lines [27–29]. Moreover, in vivo imaging studies via magnetic resonance spectroscopy and by 18F-fluorodeoxyglucose (FDG) - positron emission tomography (PET) revealed increased lactate levels and glucose uptake in the 4T1 tumors [30]. However, these studies did not investigate tumor acidosis in vivo, and the metabolic data were limited to a small tumor region; thus a comprehensive characterization of spatial heterogeneity is still lacking.

We hypothesized that imaging the spatial heterogeneity of tumor acidosis across the entire tumor by MRI-CEST pH imaging could reveal divergent invasive phenotypes. In this study, spatial tumor pH_e_ imaging was coupled with extensive *in vitro* and ex vivo characterization, including metabolism, hypoxia, and vascularization. Our results suggest that greater spatial heterogeneity of extracellular acidification is associated with increased metabolic plasticity and a more aggressive and metastatic breast cancer phenotype.

## Methods

### Animals

All animals were treated in accordance with the University Ethical Committee and European guidelines (directive 2010/63) and under the approval of the Italian Ministry of Health (authorization #741/2022). Fourteen 6-week-old BALB/c female mice (Charles River Laboratories Italia S.r.l., Calco, Italy) were used as hosts for the 4T1 and 67NR murine breast cancer cell lines.

### Cell lines

All the cell lines were passaged weekly under standard incubation conditions at 37 °C and 5% CO_2_ and tested negative for mycoplasma contamination via PCR assays.

Both cell lines were derived from a spontaneous BALB/c mammary carcinoma. Specifically, 4T1 cells were purchased from American Type Culture Collection (ATCC LgC Standards, Italy) and cultured in RPMI-1640 ATCC-modification medium (Thermo Fisher Scientific #A1049101) supplemented with 10% of fetal bovine serum (FBS), 100 U/mL penicillin and 100 µg/mL streptomycin (Pen/Strep), and 2 mM L-glutamine. 67NR cells (provided by the Cell Factory of the Molecular Biotechnology Center, Turin, Italy) were cultured in DMEM GlutaMAX (Thermo Fisher Scientific, #61965-026) supplemented with 10% FBS and 100 U/mL and 100 µg/mL Pen/Strep.

### Animal experiments

Six-week-old BALB/c female mice were orthotopically inoculated with 4T1 (1 × 10^6^ cells, *n* = 7) and 67NR cells (5 × 10^5^ cells, *n* = 7) resuspended in 50 µL of phosphate saline buffer (PBS, Sigma Aldrich, Milan, Italy) in both 4th mammary gland fat pads (to reduce the overall number of mice) under the guidance of an echographic system (FUJIFILM VisualSonic, Amsterdam, The Netherlands). Tumor growth was monitored and measured with a caliper three days per week. Tumor growth rate (TGR) was calculated by subctracting the tumor volumes at two different time points and dividing for the time difference in days. Before the MRI acquisition, mice were anesthetized via intramuscular injection of a mixture of 5 mg/kg of xylazine (Rompun, Bayer, Italy) and 20 mg/kg of tiletamine/zolazepam (Zoletil 100, Virbac, Italy), and a 27-gauge needle was introduced into the tail vein for contrast agent injection. During acquisition, the breath rate was monitored by an air pillow placed below the animal (SA Instruments, Stony Brook, USA). CEST-pH mapping and DCE-MRI were performed upon intravenous injection of 4 g I/kg body weight (b.w.) of iopamidol (Isovue370) and 0.1 mmol Gd/kg b.w. of gadoteridol (ProHance, both kindly provided by Bracco Imaging SpA, Colleretto Giacosa, Italy).

### MRI-CEST acquisition and analysis

MR images were acquired with a Bruker 7T Avance NEO 300 MRI scanner (Bruker Biospin, Ettlingen, Germany) using a 30-mm 1 H quadrature coil. Anatomical T_2w_ images were acquired to cover the whole tumor volume with a fast spin-echo turbo-rare RARE sequence with the following parameters: repetition time (TR) = 4000 ms, echo time (TE) = 5.8 ms, number of slices = 8, slice thickness = 1.5 mm, FOV = 30 mm; matrix = 256 × 256, two averages, acquisition time = 3 min, and the same geometry was used for CEST and DCE-MRI acquisitions.

Z-spectra of CEST-MRI were acquired using a single-shot RARE sequence with centric encoding (typical setting TR/TE = 12 s/3.76 ms) preceded by a 3 µT cw block presaturation pulse and by a fat-suppression module. A series of 46 MR frequencies were saturated to acquire a CEST spectrum in the frequency offset range ± 10 ppm. We used an acquisition matrix of 96 × 96 reconstructed to 128 × 128 for a field of view of 3 × 3 cm^2^ (in-plane spatial resolution = 234 μm) with 8 slices to cover the whole tumor with slice thickness = 1.5 mm [31]. MRI-CEST image acquisition was repeated before and after i.v. injection of the iodinated contrast media (dose = 4 g Iodine/kg b.w., ca. 250 µL). The total scan time was 20 min.

All the CEST images were analyzed using a custom script implemented in MATLAB (Mathworks, Inc., Natick, MA, USA) by an experimenter who was blinded to the experimental cohorts. The Z-spectra were interpolated, on a voxel-by-voxel basis, by smoothing splines, B0-shift corrected and difference contrast maps (ΔST%) were calculated by subtracting the ST contrast after iopamidol injection from the ST contrast before the injection on a per voxel basis to reduce the confounding effect of the endogenous contributions. pH_e_ values were estimated in vivo by applying the ratiometric procedure and calculated tumor pH_e_ maps were superimposed onto the anatomical reference image [32]. The acidity score was calculated to assess the heterogeneous pH_e_ distribution within the tumor regions, by subgrouping the calculated pH_e_ values in three groups, from neutral to mild acidic (pH_e_ 7.4-7.0), moderate acidic (pH_e_ 6.7-7.0) and highly acidic (pH_e_ 6.0-6.7) [12]. The overall acidity score can range from 1 (less acidic) to 3 (more acidic), defining tumor regions with different acidosis levels.

### DCE-MRI acquisition and analysis

The DCE-MRI dynamic protocol was applied after the CEST acquisition by acquiring 60 T1w images (FLASH sequence, TR/TE = 58 /1.82 ms, FA = 30°), before and after gadoteridol injection. The DCE-MRI dynamic protocol consisted of the initial acquisition of axial T_1_ maps with the variable flip angle (VFA) method by acquiring T_1w_ images with a FLASH sequence (TR/TE = 58 ms/1.82 ms, number of slices = 8, slice thickness = 1.5 mm, FOV = 30 mm, matrix = 128 × 128) by varying the flip angle (FA) at the following values: 5°, 10°, 15°, 30°, 45°, 60°. The same FLASH sequence was used with the FA set at 30°, by acquiring six initial pre-contrast images, followed by the injection of gadoteridol (ProHance, kindly provided by Bracco Imaging, Milan, Italy) through the tail vein catheter (dose 0.1 mmol Gd / kg b.w, ca. 50 µL of a 50 mM solution). After the injection, 54 dynamic post-contrast T1w images were acquired. The total scan time was 13 min. All the DCE-MRI images were analyzed using an in-house developed software in C + + code implementing MITK (http://www.mitk.org/MITK), ITK, and VTK libraries for the quantification of the pharmacokinetic parameters by applying the extended Tofts’ model [33, 34].

### Histological analyses

After MRI sessions, mice were euthanized, and the mammary tumors were excised. For the ex vivo analyses, tumors were divided in two parts: one half of the tumor was exploited for histological analyses such as IHC and IF assays, and the remaining one half of the sample was frozen at -80 °C and then used for WB assays.

To detect ex vivo hypoxic tumor regions, mice (*n = 7* for 4T1 tumors and *n* = 7 for 67NR tumors) were injected into the tail vein with 60 mg/kg (ca. 150 µL) of a 10 mg/mL pimonidazole solution (HypoxyprobeTM-1 Omni Kit, Middlesex Turnpike Burlington, MA, USA) 1 h before sacrifice. The tumor samples were both fixed in 10% PBS-buffered formalin and in optimal cutting temperature matrix compound (Tissue-Tek^®^ OCT™) and stored at -80 °C.

The presence of pulmonary metastasis was assessed by histological examination. Immediately after mice sacrifice, lungs were resected and fixed in phosphate-buffered 4% paraformaldehyde and paraffin-embedded. Histological sections were cut from all lobes at 100 μm intervals and were stained for hematoxylin and eosin. Pulmonary metastases were observed and counted with an optical microscope (DM6. Leica Microsystems, Wetzlar, Germany) and ImageJ software (US National Institutes of Health, Bethesda, MD, USA). Groups of five or more cells were scored as metastasis.

### Immunofluorescence microscopy

Frozen tumor slices (5-µm thickness) were fixed with cold acetone for 10 min and dried in air for 30 min. The slices were rinsed with PBS, permeabilized with PBS + Triton X100 0.1% (Sigma #T8787) and blocked with 10% goat serum for 1 h at room temperature (RT). The slices were then incubated with rat anti-CD31 (1:200, BD Pharmigen, #557355), rabbit anti-LAMP2a (1:500, Abcam, ab18528), and rabbit anti-pimonidazole (1:100, Hypoxyprobe^TM^-1 Omni Kit, MA, USA) primary antibodies overnight at 4 °C and visualized using goat anti-rat (1:500, Alexa Fluor 568, Invitrogen) and goat anti-rabbit (1:500, Alexa Fluor 488, Invitrogen) secondary antibodies. Nuclei were stained with DAPI (1:1000).

All the immunofluorescence staining were analyzed using a ViCO system microscope (Nikon) and the fluorescent signal was quantified using the ImageJ software (https://imagej.net/ij/index.html); the fluorescence intensity was quantified as the area of fluorescence measured in 10 pictures for each tumor, acquired with the same parameters and analyzed with the same threshold [35].

### Immunohistochemistry

FFPE blocks were cut 5 μm thick and slices were heated in the stove at 60 °C and then deparaffinized and re-hydrated according to standard protocol. After tissue rehydration, antigen retrieval was performed in a water bath at 96 °C for 15 min with citrate buffer at pH 6.0 (Abcam, ab93678), then tissue sections were permeabilized with PBST 0,1% and then endogenous peroxidase was blocked with 3% of H_2_O_2_. Blocking was performed with 10% of NGS for 1 h at RT. Tissue sections were incubated with primary antibodies: rabbit anti-LDHA (1:100, Invitrogen, #PA5-27406), rabbit anti-PDK1 (1:200, Invitrogen, #MA5-32702), and rabbit anti-CAIX (1:200, Novus Biological, NB100-417) overnight at 4 °C. The next day, the sections were incubated with a goat anti-rabbit HRP secondary antibody (1:500, Abcam, ab6721) for 1 h at RT. Tissue sections were then incubated with fresh DAB chromogen staining (Sigma #D3939). Sections were then dehydrated with increasing % alcohols and slides mounted with coverglass.

To assess the spatial distribution of the investigated markers, a semiquantitative score was exploited by scoring the sections for both the intensity of staining (0, no staining; 1, weak; 2, mild; 3, strong staining) and for the covered area (0, absence of labeling; 1 for 25–50% of covered area; 2 for 50–75%; 3 for 75–100% of covered area) by diving the tumor boundaries in the core and in the rim regions. Multiplication of both scores allowed the final score ranging from 0 to 9 [36].

### Western blot

Protein extraction was performed using RIPA lysis buffer (Merck Millipore #20–188), supplemented with a protease inhibitor cocktail (Sigma #P2714). Protein concentrations were measured with a Thermo Fisher Pierce BCA Protein Assay Kit (Thermo-Fisher, #23225), and 30 µg of total protein were separated with a Bio-Rad Mini-PROTEAN^®^ TGX TM Gel (Bio-Rad #456–9034). Proteins were transferred to a 45-µm-pore polyvinylidene difluoride (PVDF) membrane (Immobilon PSQ, Millipore) and the membranes were blocked with 5% milk. Primary antibodies for LDH-A, PDK1, CAIX (1:3000; #PA5-27406, #MA5-32702, NB100-417, respectively), an optimized cocktail of antibodies for the electron transport chain (ETC) complexes (total OXPHOS, 1:1000; Abcam #ab110413), and β-actin (1:3000; Sigma-Aldrich #A1978) were detected by anti-rabbit IgG (1:5000; Sigma # A6154) and anti-mouse IgG (1:5000; Sigma #A4416). Signals were detected with Pierce TM ECL western Blotting Substrate kit (Thermo-Fisher #32106) and subsequent bands were quantified with the ImageLab software (Bio-Rad Laboratories S.r.l., Segrate, Italy).

### Seahorse XFe96 metabolic assays

Seahorse XF assays were performed as previously described [37]. Briefly, 2.5–3.5 × 10^4^ cells were seeded in XFe96 cell culture plates and then subjected to the extracellular flux (XF) glycolytic rate assay and the XF Mito Stress test (Agilent Technologies, Santa Clara, CA, USA) following the manufacturer’s instructions. Importantly, the extracellular acidification (ECAR) and the amount of oxygen consumed (OCR) by the cells, parameters indicative of fermentative and oxidative metabolism, were monitored in real-time after the ATP synthase inhibitor oligomycin, proton uncoupler carbonyl cyanide p-(trifluoromethoxy) phenylhydrazone (FCCP), respiratory complex I/III inhibition using a mix of Rotenone/Antimycin A (Rot/AA) and/or the glycolysis inhibitor using 2-deoxyglucose (2-DG). Protein quantification was used to normalize the results.

### Radiolabeled glucose uptake assay

Breast cancer cells were seeded into a 12-well plate under basal culture conditions. Glucose uptake was evaluated by incubating the cells with a buffered solution (140 mmol/L NaCl, 20 mmol/L HEPES/Na, 2.5 mmol/L MgSO_4_, 1 mmol/L CaCl_2_, and 5 mmol/L KCl, pH 7.4) containing ^14^C-glucose (Perkin Elmer) for 15 min at 37 °C. The cells were subsequently washed with cold PBS and lysed with 0.1 M NaOH. The incorporated radioactive glucose-derived signal was measured by liquid scintillation counting and normalized to the protein content.

### Oroboros O2k-FluoRespirometer

Oxygen consumption was analyzed in 2 mL glass chambers at 37 °C using the Oroboros oxygraph-2 K high-resolution respirometer (Oroboros Instruments, Innsbruck, Austria), and the substrate, uncoupler, inhibitor, and titration (SUIT) 003 O2 ce D009 protocols [38] were used, as previously described [29]. After instrumental air calibration, 1 × 10^6^ cells resuspended in a complete culture medium were analyzed. The oxygen flux normalized to the cell number was calculated as the negative time derivative of the oxygen concentration, measured in sealed chambers, and normalized to the instrumental background (measured in a dedicated experiment before the cells were assayed). The parameters that are measured are the basal respiratory activity (R), the nonphosphorylating state of uncoupled respiration due to proton leakage, proton and electron slip, and cation cycling [39] (L), the maximal capacity of oxygen utilization (E) and the residual oxygen consumption (ROX). Data acquisition and analysis were performed using DatLab software (Oroboros Instrument) and the oxygen fluxes recorded in the individual titration steps were corrected for ROX.

### Confocal image acquisition

Glass coverslip (Nunc LabTek^®^ Chambered CoverGlass, Thermo Scientific)-plated cells were labeled with 100 mM MitoTraker™ Green (Thermo Fisher Scientific #M7514) to reveal the number of mitochondria and 100 mM tetramethylrhodamine, ethyl ester and perchlorate (TMRE) (Thermo Fisher Scientific, T669) for 15 min at 37 °C to reveal the mitochondrial membrane potential. For nuclear staining, the cells were incubated with Hoechst 33,342 solution (Thermo Fisher Scientific #62249) for 15 min at 37 °C. All fluorescence samples were examined at RT using a microscope (TCS SP8, Leica Microsystems, Wetzlar, Germany). Images were captured using the Leica LAS-AF image acquisition software.

### Flow cytometry analysis

Breast cancer cells (8 × 10^4^ cells/well) were seeded into 12-well plates. The next day, the cells were stained with 100 mM MitoTracker™ Green (Thermo Fisher Scientific #M7514) for 15 min at 37 °C. Live cells were resuspended in PBS supplemented with 0.1% FBS and subjected to flow cytometry analysis using a FACSCanto II (BD Bioscience). A total of 1 × 10^4^ cells were analyzed for the median fluorescence intensity (MFI) of the probe.

### In silico analysis

Transcriptomic data of 67NR and 4T1 cells were retrieved from the GSE236033 database that we have previously published [29]. Briefly, the transcriptomic data were derived from 67NR or 4T1 cells that were inoculated into the mammary fat pad of Ub-GFP mice. Total RNA was extracted from isolated FACSorted GFP-negative, CD45-negative tumor cells and subjected to Illumina MouseWG-6 v2.0 expression beadchip. The data were normalized using quantile normalization with IlluminaGUI in R.

### Statistical analysis

Statistical analysis was performed using GraphPad Prism 8 (GraphPad Software, San Diego, CA, USA). Unless stated otherwise, all numerical data are expressed as the mean ± standard error of the mean (SEM) or standard deviation (SD). All experiments were conducted at least 3 times independently, with 3 or more technical replicates for each experimental condition tested. Comparisons between two groups were made via two-tailed, unpaired Student’s t-test. Statistical significance was defined as follows: * *p* < 0.05; ** *p* < 0.01; *** *p* < 0.001, **** *p* < 0.0001; when differences were not statistically significant or the comparison was not biologically relevant, no indication was reported in the figures. ROC curves were plotted to assess the performance of the MRI-CEST tumor pH_e_ metrics in differentiating 4T1 tumors from 67NR tumors. The accuracy, sensitivity, specificity, and AUC were also calculated.

### Data availability

The data generated in this study are available upon request from the corresponding author.

## Results

### *In vitro* characterization of 4T1 and 67NR cells reveals different acidosis and metabolic features

**Fig. 1:**
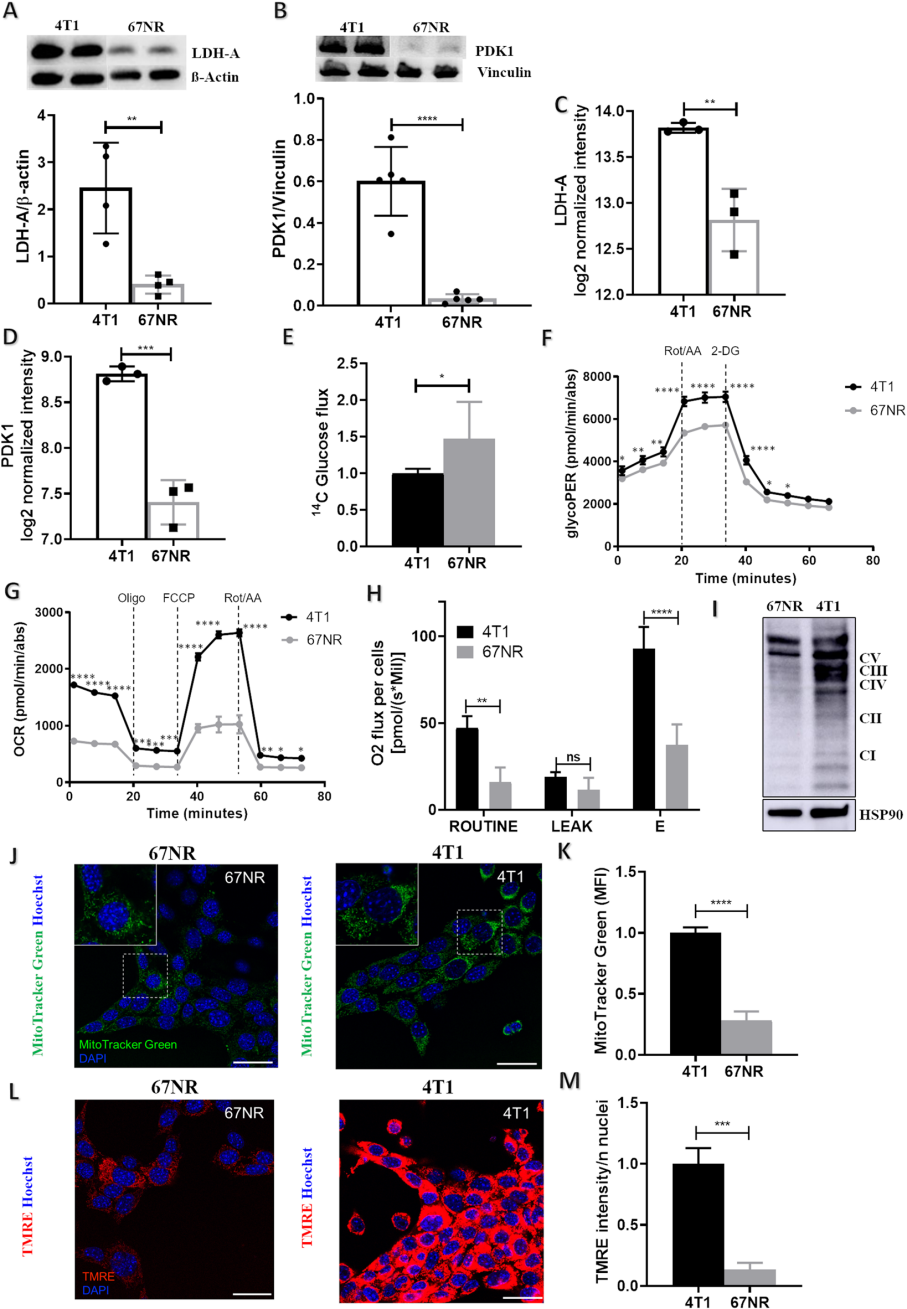
*In cellulo* characterization of energy metabolism in 4T1 and 67NR cells. **A, B**) Protein quantification of (**A**) LDHA and (**B**) PDK1 assessed by western blotting. **C, D**) Gene expression levels of (**C**) LDH-A and (**D**) PDK1, *n* = 3 biological replicates. (**E**) ^14^C-glucose uptake measured in 67NR and 4T1 cells. The relative upload capacity is shown using metastatic cells as the comparator. Data represent means ± SEMs. *n* = 3 biological replicates in at least technical triplicate. Student’s t-test. **F**) Seahorse XFe96 glycolytic rate assay performed in 4T1 and 67NR cells subjected to serial injections of the respiratory complex I inhibitor rotenone together with the respiratory complex III inhibitor antimycin A (Rot/AA, 0.5 µM) and 2-deoxyglucose (2-DG, 50 µM). The glycolytic proton efflux rate (glycoPER) was calculated in real-time. Data represent means ± SEMs and are normalized on protein content. *n* = 3 biological replicates in at least either duplicate or technical triplicate. Two-way ANOVA, Tukey’s correction. **G**) Seahorse XFe96 Mito Stress Test performed in 4T1 and 67NR cells in the presence of standard condition (full medium). The oxygen consumption rate (OCR) was calculated in real-time after the administration of the ATP synthase inhibitor oligomycin (Olygo, 1.5 µM), the proton uncoupler carbonyl cyanide p-triflouromethoxyphenylhydrazone (FCCP, 1 µM), and a mixture of rotenone and antimycin A (Rot/AA, 0.5 µM). Data represent means ± SEMs and are normalized on protein content. *n* = 3 biological replicates in at least either duplicate or technical triplicate. Two-way ANOVA, Tukey’s correction. **H**) TNBC cells subjected to high-resolution respirometry analysis by Oroboros-O2K instrument. Bar chart graphs of basal oxygen consumption (ROUTINE), proton leak (LEAK), and maximal oxygen consumption (**E**) values subtracted from residual oxygen consumption (ROX) in 4T1 and 67NR cells are shown. Data represent means ± SEMs. *n* = 3 biological replicates. Student’s t-test. **I**) Western blot analysis for the electron transport chain (ETC) complexes (CI-V, total OXPHOS). **J, K**) TNBC cells subjected to confocal (**J**) and cytofluorimetric (**K**) analyses. Representative pictures of MitoTracker green-stained cells are shown (green: mitochondria; blue: Hoechst, nuclei). The data represent means ± SEMs. *n* = 3 biological replicates. Student’s t-test. **L, M**) 4T1 and 67NR cells were subjected to TMRE staining by confocal microscopy. Representative images are shown (**L**, red: active mitochondria; blue: Hoechst, nuclei). The TMRE intensity was quantified as described in the Methods section. Data represent the means ± SEMs. *n* = 3 biological replicates. Student’s t-test (**M**). * *P* ≤ 0.05; ** *P* ≤ 0.01; *** *P* ≤ 0.001; **** *P* ≤ 0.0001

Tumor acidosis promotes cancer aggressiveness and metastatic potential and is influenced by the metabolic properties of the tumor. By using two cellular triple-negative models derived from the same primary murine breast adenocarcinoma but with different metastatic capacities, we investigated whether there are differences in acidosis and metabolic features between the non-metastatic 67NR cells and the overtly metastatic 4T1 cells and whether acidosis is related to and influences cancer cell metabolism in breast cancer. Notably, our previous gene set enrichment analysis (GSEA) [29] revealed a positive association between the HALLMARK GLICOLYSIS gene set (M5937, NES:2.9; FDR q-value = 0.001) and the 4T1 transcriptomic profile, suggesting a potential enhancement of the glycolytic pathway in the 4T1 cells compared with that in 67NR cells. To characterize whether differences in glucose metabolism occur between the 4T1 and 67NR cell lines cultured *in vitro*, we first performed gene expression and protein analyses of key glycolytic-related enzymes. Lactate dehydrogenase A (LDH-A), known to promote the fermentative pathway and lactate production, and pyruvate dehydrogenase kinase (PDK-1), which inhibits the pyruvate dehydrogenase complex and the pyruvate entrance into the TCA cycle, were found to be overexpressed in 4T1 cells compared with 67NR cells at protein levels (Fig. 1A, B). Crucially, these data were confirmed at mRNA level on ex vivo derived 67NR and 4T1 cells retrieved from our previously published transcriptomic analysis (Fig. 1C, D).

Since increased glucose uptake may be a prerogative feature of cells that exploit glucose-dependent fermentative catabolism, we then investigated whether the increased expression levels of LDH-A and PDK-1 were paralleled by enhanced glucose upload and subsequent glucose-dependent metabolization of ^14^C-labeled glucose in the highly metastatic model, a trait of the so-called Warburg metabolism. Unexpectedly, the radioactive tracing analysis did not confirm this hypothesis (Fig. 1E). However, when cells were subjected to real-time measurements of the extracellular acidification rate (ECAR) and oxygen consumption rate (OCR) by Seahorse analyses, 4T1 cells appeared more metabolically active (i.e., “energetic”) than 67NR cells did. Indeed, a Glycolytic Rate Assay, that discriminates between the ECAR generated by mitochondrial-derived CO_2_ and that generated by glycolysis, revealed that 4T1 cells enhanced the glycolytic Proton Efflux Rate (glycoPER, Fig. 1F) measured following mitochondrial respiratory inhibition using a mixture of Rotenone and Antimycin A (Rot/AA) and glycolysis inhibition using 2-deoxyglucose (2-DG). Accordingly, basal and compensatory glycolysis were increased in the more aggressive cell line compared with the less invasive 67NR line (Suppl. Figure 1 A, B). Concomitantly, the Mito Stress Test revealed increased OCR in 4T1 cells under metabolic stress conditions (i.e., serial injections of oligomycin, Oligo, FCCP, Rotenone/Antimycin A, Rot/AA, Fig. 1G). In line, their basal and maximal respiration was enhanced compared to 67NR cell line (Suppl. Figure 1 C, D), supporting recent studies on the metabolism of 4T1 and 67NR cells that highlighted the greater metabolic plasticity of 4T1 cells [28].

Oxygen consumption real-time monitoring via an Oroboros oxygraph-2K high-resolution respirometer confirmed the metabolic profiles derived by Seahorse analysis, as shown by enhanced basal (ROUTINE) and maximal (E) respiration in the 4T1 cells (Fig. 1H). Increased mitochondrial activity and/or mass were confirmed by Western blot analysis, which revealed greater protein levels of the electron transport chain mitochondrial complexes I, II, and IV in the 4T1 cells than in 67NR cells (Fig. 1I), a trait that was paralleled by increased levels of the mitochondrial mass and increased mitochondrial membrane potential (assayed using confocal and FACS analysis of the fluorescent dyes MitoTracker Green and TMRE) (Fig. 1J-M).

Together these data highlight that the 4T1 cells can exploit fermentative and oxidative metabolism to a greater extent than can 67NR cells, a trait that could be associated with their increased invasive potential and is in line with the high metabolic plasticity previously described for the 4T1 cells [29].

### In vivo MRI-CEST pH imaging reveals strong differences in tumor extracellular acidity between the two syngeneic breast tumor models

**Fig. 2:**
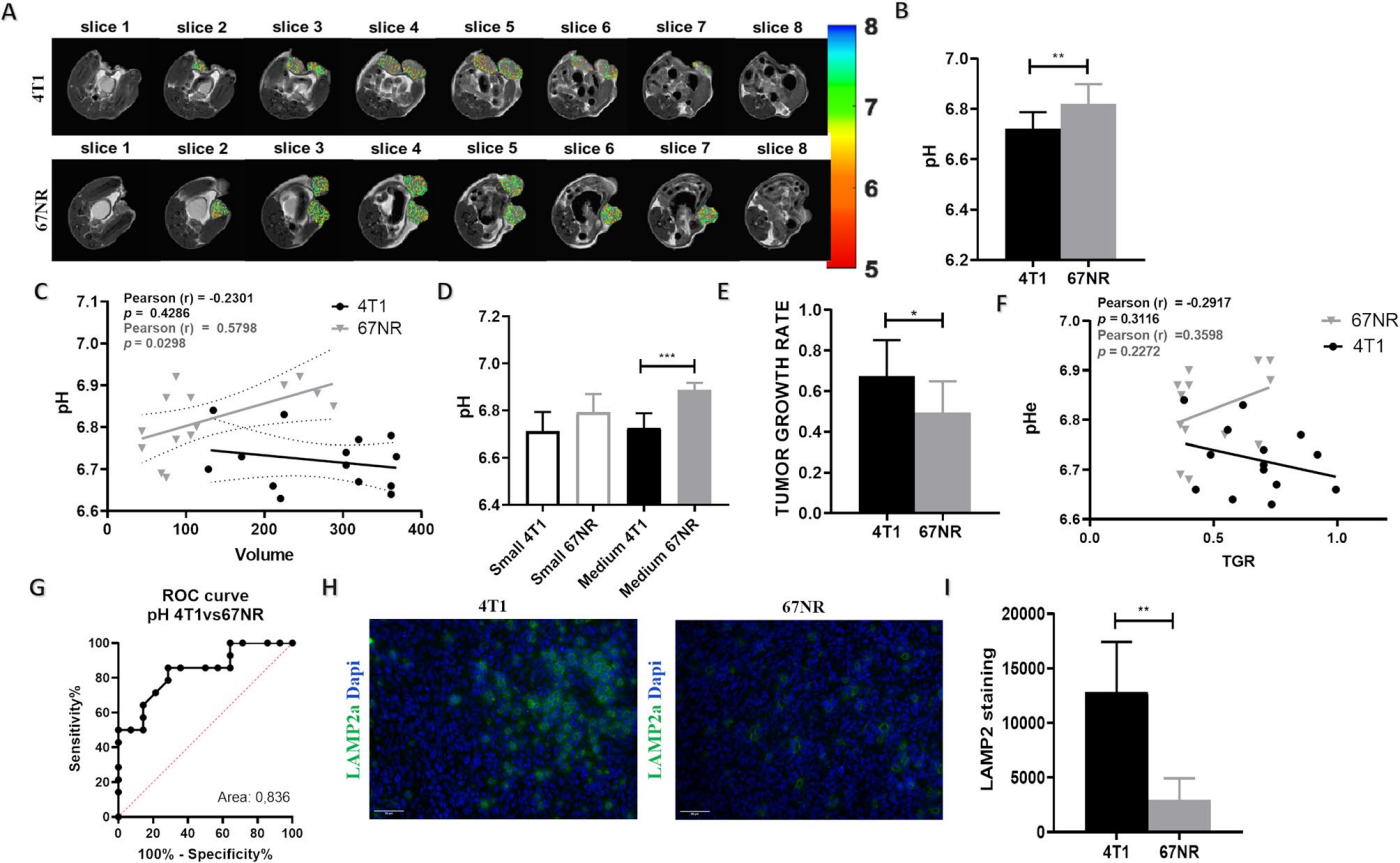
Imaging results from CEST-MRI. (**A**) Iopamidol extracellular pH maps of representative patients, reporting more acidic pH_e_ values (orange and yellow pixels) in 4T1 tumors (top) and less acidic pH_e_ values (more green pixels) in 67NR tumors (bottom). Bar graphs of (**B**) pH_e_ values of 4T1 and 67NR tumors. (**C**) Correlation between tumor volume and pH_e_ calculated via CEST-pH imaging. (**D**) Correlation between pH_e_ and different tumor volumes (small < 220 mm^3^ and medium > 220 mm^3^). The data represent means ± SDs. (**E**) Bar graph of TGR of 4T1 and 67NR tumors. (**F**) Correlation between tumor pH_e_ calculated via CEST pH imaging and TGR. (**G**) ROC curve comparisons for distinguishing between metastatic and non-metastatic tumors in terms of tumor pH_e_. (**H**) Immunofluorescence staining of LAMP2 (*n* = 4 biological replicates). (**I**) Quantification of the positive fluorescent area in 4T1 and 67NR tumor samples. The data represent the means ± SDs. ** *P* ≤ 0.01; *** *P* ≤ 0.001

Exposure to an acidic tumor microenvironment contributes to creating harsh microenvironmental conditions that select for a more aggressive and invasive phenotype. Given the different metastatic potentials of the two cell lines, we assessed the spontaneous lung metastasis arising from the primary 4T1 and 67NR breast tumors. Whereas the 4T1 tumor-bearing mice presented clear evidence of several lung metastases, lung nodules were almost absent in the mice with 67NR tumors (Suppl. Figure 2). We hypothesized that this divergent invasive phenotype was associated with increased extracellular acidity and measured the in vivo tumor acidosis with MRI-CEST pH-imaging upon intravenous administration of the pH-responsive Iopamidol contrast agent in 4T1 and 67NR tumor-bearing mice. The calculated tumor pH_e_ maps (Fig. 2A) revealed more acidic pH_e_ values in the 4T1 tumors, whereas the 67NR tumors presented less-acidic pH_e_ values (mean pH_e_ values of 6.72 ± 0.06 and 6.81 ± 0.07, for the 4T1 and 67NR tumors, respectively; *p* < 0.01, Fig. 2B). The correlation between tumor volume and pH_e_ showed an opposite trend between the two groups: while 67NR tumors presented a moderate correlation with less acidic pH_e_ values during tumor progression, 4T1 tumors did not show any correlation between pH_e_ values and volume (r^2^ = 0.58, *p* < 0.05 for the 67NR tumors, r^2^ = -0.23, *p* = 0.43 for the 4T1 tumors; Fig. 2C). To further investigate differences in tumor acidosis based on tumor size, samples were further subdivided into small (< 200 mm^3^) and medium (> 200 mm^3^) sizes. We observed that medium-sized 4T1 tumors had statistically significant lower pH_e_ values than 67NR tumors did (6.72 ± 0.06 and 6.88 ± 0.02, respectively, *p* < 0.001, Fig. 2D) and a similar tendency was observed for small tumors, although the difference was not statistically significant.

We also considered to evaluate the tumor growth rate (TGR) that is related to metabolic rate, hence to tumor acidosis, but less dependent on the absolute tumor volume size. Interestingly, we observed a marked difference between the two breast cancer cell lines, with the 4T1 showing statistically significant larger TGR values than the 67NR tumors (TGR = 0.67 ± 0.17 and 0.49 ± 0.15, respectively, *p* = 0.0102, Fig. 2E). Additionally, relating TGR values to tumor acidosis, we observed an inverse trend between the two tumor types but with weak correlations, with 4T1 tumors showing increased extracellular acidification with larger TGR values, whereas the 67NR tumors showed a decrease in tumor acidosis for larger TGR values (r^2^ = -0.292, *p* = 0.312 and r^2^ = 0.360, *p* = 0.227 for 4T1 and 67NR tumors, respectively, Fig. 2F).

The receiver operating characteristic analysis revealed a good diagnostic performance for the extracellular tumor pH metric for distinguishing between the more and the less-metastatic tumor types (Fig. 2G; Table 1).

**Table 1:** ROC curve analysis

Quantitative indicators	AUC (95% CI)	*p*	Sensitivity	Specificity	Youden index
Tumor pHe	0.837 (0.649–0.948)	< 0.0001	0.714	0.857	0.571
Acidity score	0.834 (0.646–0.947)	< 0.0001	0.786	0.857	0.643

AUC, area under the curve; CI, confidence interval

Previous studies have shown increased expression of LAMP2 (lysosomal-associated membrane protein 2 A) in tumor tissues, reflecting a link with acid adaptation [40]. LAMP2 was evaluated ex vivo via immunofluorescence and was found to be significantly increased in 4T1 tumors than in 67NR ones (Fig. 2H, I), which can be related to the CEST results for tumor pH_e_ and may be associated with increased tumor aggressiveness.

### Spatial intratumoral heterogeneity of acidosis enables differentiation between high- and low- malignancy tumors

Although acidosis has been linked to increased invasiveness, in vivo studies on its spatial distribution are lacking. Therefore, we explored whether varying spatial distributions of pH_e_ values within tumors are correlated with increased heterogeneity and, consequently, invasiveness.

**Fig. 3:**
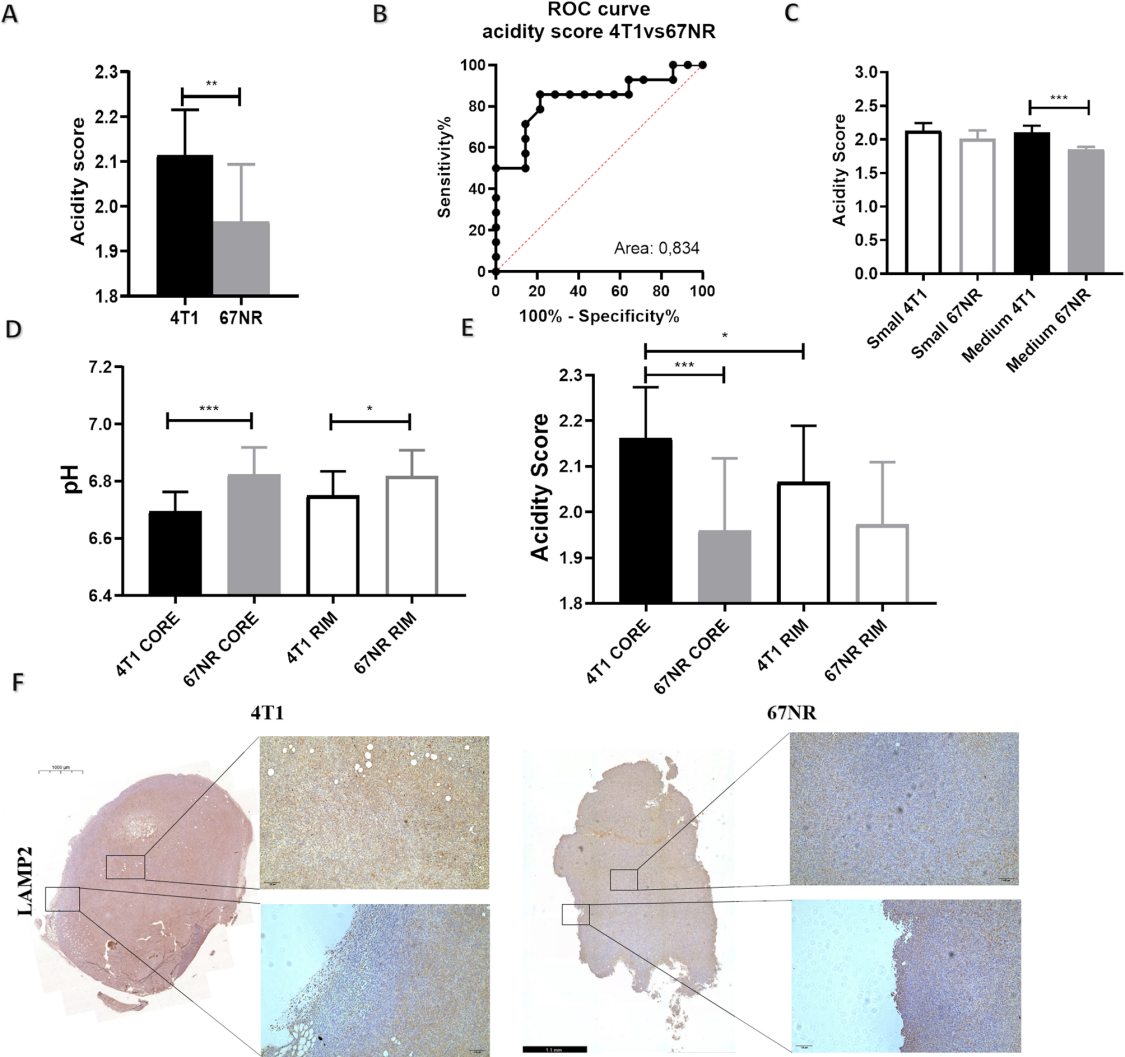
In vivo and ex vivo characterization of tumor heterogeneity. (**A**) Bar graph of the acidity score as a metric of pH_e_ heterogeneity. (**B**) ROC curve comparison of the acidity score for distinguishing between metastatic and non-metastatic tumors. (**C**) Bar graph of acidity score values with different tumor volumes (small < 220 mm^3^ and medium > 220 mm^3^). Bar graphs of (**D**) tumor pH_e_ and (**E**) acidity score value distribution in 4T1 and 67NR tumors according to subregion analysis of the rim and core regions. (**F**) IHC analysis of LAMP2 expression and distribution of signals through rim and core regions in 4T1 and 67NR representative patients (*n* = 4 biological replicates). The data represent the means ± SDs. * *P* ≤ 0.05; ** *P* ≤ 0.01; *** *P* ≤ 0.001

First, intratumoral pH_e_-heterogeneity was assessed by the acidity score, a metric that calculates the heterogeneity of the distribution of pH_e_ values, with values close to 3 indicating a greater degree of heterogeneity, whereas values close to 1 indicating lower heterogeneity [12]. The acidity score values highlighted a marked heterogeneity of pH_e_ values within the 4T1 tumors compared with the 67NR tumors, with mean values of 2.11 ± 0.10 and 1.96 ± 0.12, respectively (*p* < 0.01, Fig. 3A). The calculated ROC curve for the acidity score metric showed comparable diagnostic performance to that of the average pH_e_ metric in distinguishing between the two tumor types (Fig. 3B). To investigate whether there were differences in tumor heterogeneity based on tumor size, we calculated the acidity score values for small- and medium-sized tumors and observed that medium-sized 4T1 tumors exhibited significantly greater heterogeneity than 67NR tumors did (2.10 ± 0.09 and 1.85 ± 0.03, respectively, *p* < 0.001, Fig. 3C). The relationship between TGR and acidity scores showed an opposite trend with a moderate relationship, with 4T1 tumors showing higher acidity scores with increasing TGR values, whereas 67NR tumors show the opposite trend (r^2^ = 0.319, *p* = 0.266 and r^2^ = -0.421, *p* = 0.152 for 4T1 and 67NR tumors, respectively, Suppl. Figure 3).

Additional quantification of the spatial heterogeneity was performed by dividing tumors into two sub-regions: the “rim”, referring to regions closer to tumor edges, and the “core”, corresponding to inner tumor areas. The pH_e_ values of the rim and core regions of the 4T1 and 67NR tumors were then analyzed. 4T1 tumors revealed lower pH_e_ values, although not statistically significant, in the core regions compared to the rim ones (6.69 ± 0.06 and 6.74 ± 0.08, respectively, *p* = 0.0695), while 67NR ones showed no differences (6.82 ± 0.09 and 6.81± 0.08, Fig. 3D). Additionally, 4T1 rim and core regions exhibited lower pH_e_ values than 67NR ones. Similar results were obtained for the acidity score distribution, which highlighted higher values in the core regions of the 4T1 tumors than in the rim regions (2.16 ± 0.11 and 2.06 ± 0.12, respectively, *p* < 0.05). Furthermore, the 4T1 tumor core regions presented strongly greater values than the 67NR regions did (2.16 ± 0.11 and 1.95 ± 0.15, respectively). No statistical significant differences were detected between the 67NR rim and core regions (1.95 ± 0.15 and 1.97 ± 0.13, respectively, Fig. 3E). Together, these findings confirm a strong difference in tumor acidosis between the two tumor models, revealing also a marked intratumoral heterogeneity in 4T1 tumors, whereas 67NR tumors exhibit fewer differences between the rim and core regions.

Additionally, ex vivo immunohistochemistry for LAMP2 (Fig. 3F) revealed a stronger signal in 4T1 tumors within the whole section, which was also observed to be closer to necrotic areas, whereas 67NR tumors presented less signal mainly found at tumor boundaries.

### 4T1 and 67NR primary tumors present distinct vascular and hypoxic phenotypes

**Fig. 4:**
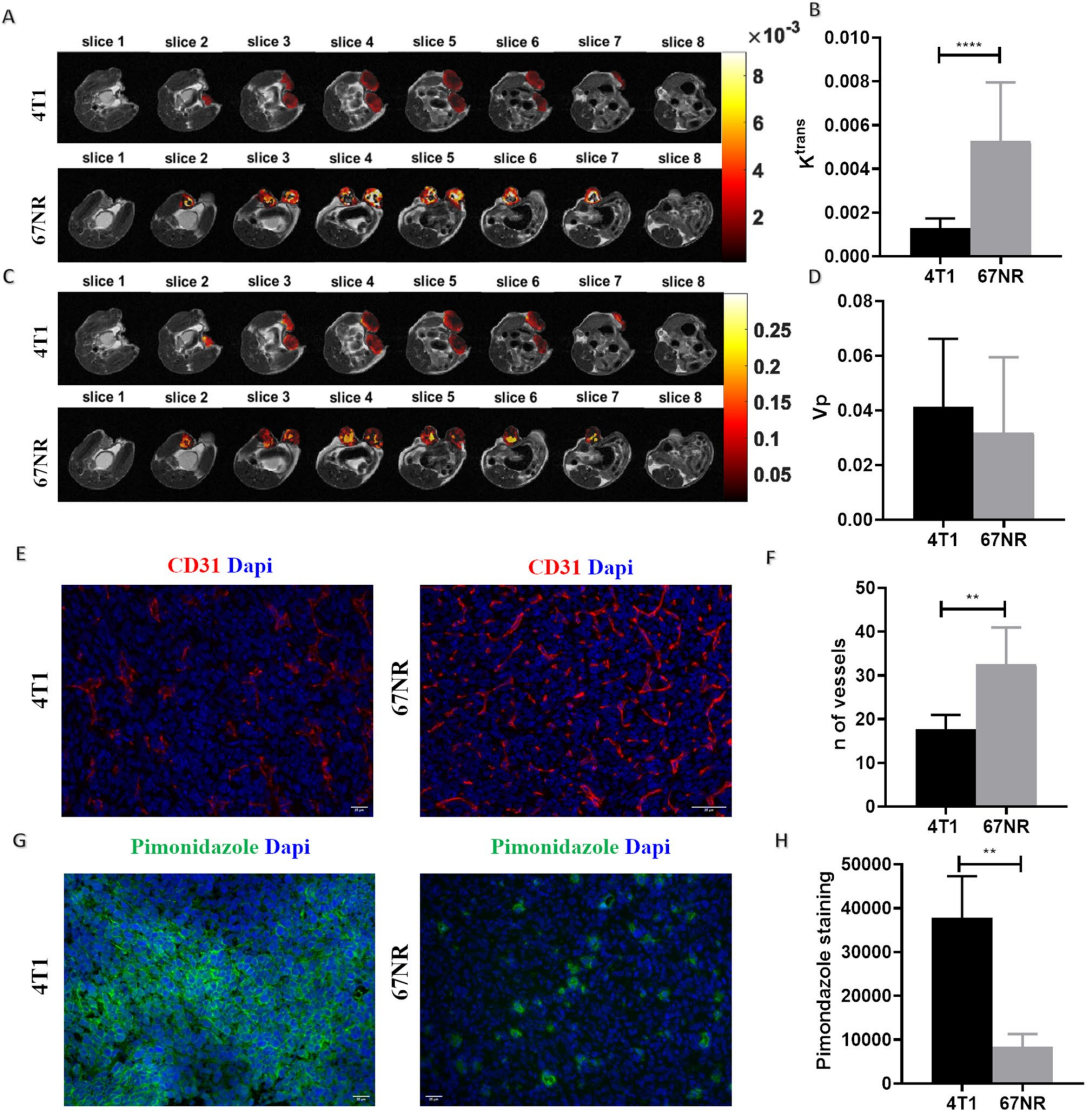
DCE-MRI results of tumor vessel permeability and perfusion and ex vivo characterization of the tumor microenvironment. (**A**) K^trans^ (permeability) parametric maps of representative patients (4T1 top, 67NR bottom). (**B**) Bar graph of K^trans^ values. (**C**) v_p_ (plasma volume fraction) parametric maps of representative patients (4T1 top, 67NR bottom). (**D**) Bar graph of v_p_. (**E**) Immunofluorescence staining of CD31 and (**F**) the number of vessels resulting from the MVD count of the 4T1 and 67NR tumor samples. (**G**) Pimonidazole staining and (**H**) quantification of the positive fluorescent area in 4T1 and 67NR tumor samples. The data represent the mean ± SDs (*n* = 4 biological replicates). ** *P* ≤ 0.01; **** *P* ≤ 0.0001

Previous studies have shown that increased metabolism and lactate production, in combination with irregular tumor vasculature and its failure to eliminate the acidic metabolites and protons accumulated in the tumor microenvironment, can contribute to tumor acidosis [8]. For this reason, we characterized in vivo tumor vessel permeability and plasmatic volume in 4T1 and 67NR tumor models by DCE-MRI [41]. The calculated pharmacokinetic parameter *K*^*trans*^ provided information related to tumor vessel permeability and the parametric maps (Fig. 4A) of representative tumors highlighted a different distribution pattern: 4T1 tumors presented lower vessel permeability in the inner tumor masses than in the outer (rim) regions, whereas 67NR tumors showed higher permeability inside tumor regions. Moreover, compared with 67NR tumors, 4T1 tumors exhibited markedly lower vessels permeability (mean *K*^*trans*^ = 0.0012 ± 0.0004 and 0.0052 ± 0.0026, for 4T1 and 67NR tumors, respectively, *p* < 0.0001, Fig. 4B). On the other hand, the *v*_*p*_ values, which describe the volume fraction in a voxel occupied by blood vessels, reported comparable tumor plasmatic volumes in both tumor models (mean values of *v*_*p*_ 0.04 ± 0.02 and 0.03 ± 0.02 for 4T1 and 67NR tumors, respectively, Fig. 4C, D). Ex vivo immunofluorescence staining for CD31 as a marker of endothelial cells and average microvessel density (a-MVD) were used for a better characterization of tumor vascularization: fewer vessels were observed in 4T1 tumors than in 67NR ones (18 ± 3 and 33 ± 8 respectively, *p* < 0.01, Fig. 4E, F), which may explain the in vivo results of tumor vessel permeability and correlate with the larger necrotic areas (although more limited in number) observed in 4T1 tumors. Tumor sections were also stained for pimonidazole to evaluate tumor hypoxia: 4T1 tumors presented greater pimonidazole staining than 67NR tumors did (Fig. 4G, H), which may be correlated with the lower number of vessels and greater tumor acidosis observed in 4T1 tumors.

### Ex vivo characterization reveals different tumor metabolic features between 4T1 and 67NR breast cancers

Ex vivo characterization of metabolism and microenvironmental factors in 4T1 and 67NR tumor samples was conducted to validate the in vivo imaging-based findings.

**Fig. 5:**
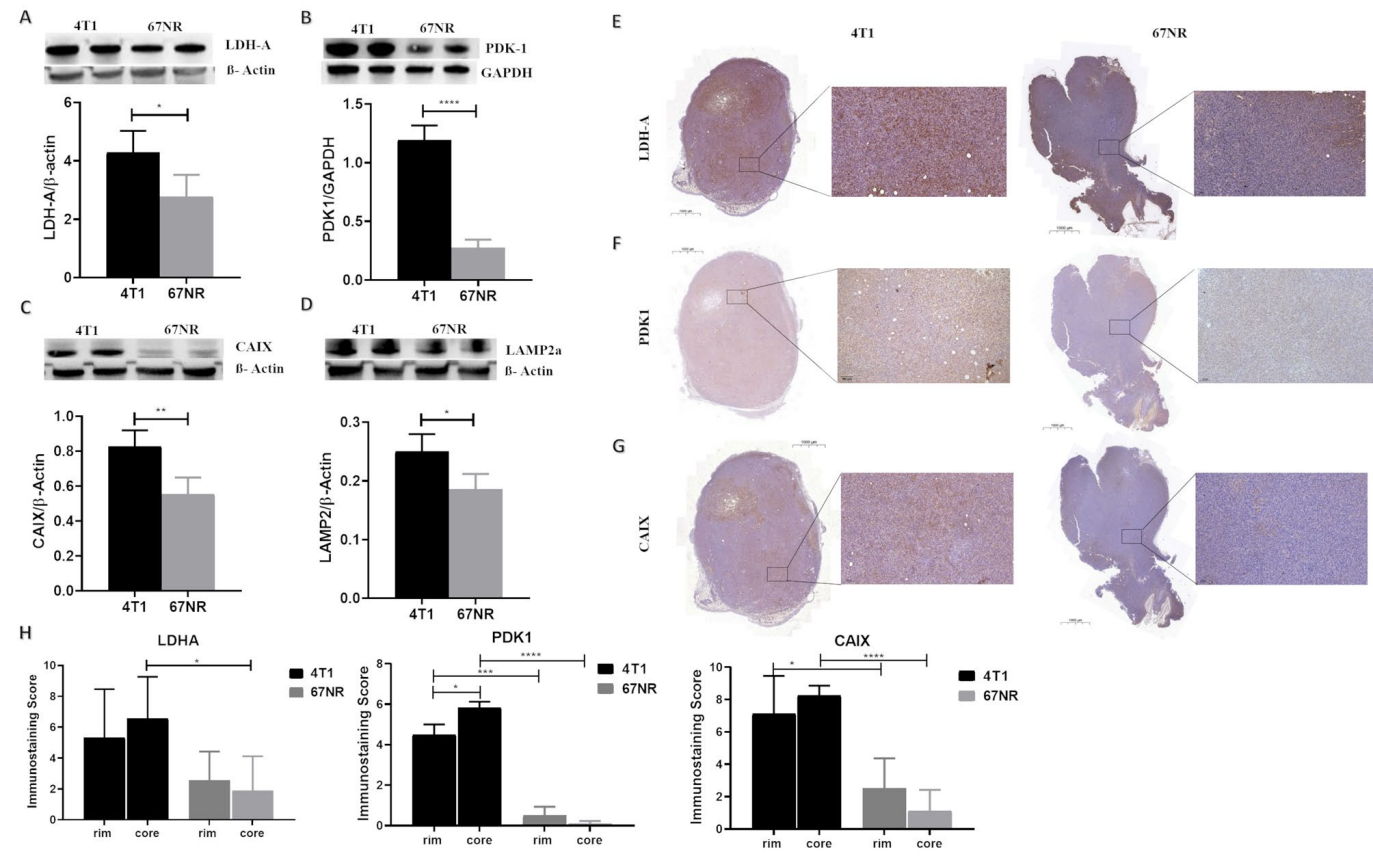
Ex vivo metabolic characterization of tumor specimens. Western blot analyses and protein quantification of (**A**) LDH-A, (**B**) PDK1, (**C**) CAIX, and (**D**) LAMP2. Immunohistochemistry of (**E**) LDH-A, (**F**) PDK1, and (**G**) CAIX in 4T1 and 67NR (left and right, respectively) specimens. (**H**) Semiquantitative analysis of the immunohistochemistry sections reporting the immunostaining score for LDH-A, PDK1 and CAIX calculated in the rim and in the core regions of the 4T1 and 67NR tumors. The data represent the mean ± SDs. * *P* ≤ 0.05; ** *P* ≤ 0.01; **** *P* ≤ 0.0001

Higher glycolytic metabolism increases the production of acidic metabolites and protons, which are responsible for the acidification of the tumor microenvironment, thus promoting cancer aggressiveness and invasive behavior. To confirm the correlation between tumor acidosis and metabolic alterations, and indeed their relationship with a malignant phenotype, we evaluated glycolysis markers such as LDH-A and PDK-1, in addition to LAMP2 and CAIX, which are involved in both tumor acidosis and hypoxia. Western blotting revealed greater LDH-A and PDK-1 expression in 4T1 tumors than in 67NR tumors (*p* < 0.05 and *p* < 0.0001, respectively, Fig. 5A, B), suggesting that aggressive and metastatic breast tumors may depend on glycolysis to sustain tumor progression. Higher CAIX and LAMP2 protein expression levels were also detected in 4T1 tumors (*p* < 0.01 and *p* < 0.05, respectively, Fig. 5C, D), which is in line with the results of pimonidazole and LAMP2 IF staining, thus confirming the observed in vivo results on tumor acidosis.

Immunohistochemical staining allowed us to better understand the spatial distribution of each metabolic marker within tumor sections in both tumor models. We observed that LDH-A staining in 4T1 tumors was more intense and jeopardized inside the whole tumor section, whereas 67NR tumors presented lower LDH-A expression (Fig. 5E). The tumor sections were also stained for PDK1 and CAIX, which revealed higher expression of both markers in the 4T1 tumors in contrast to 67NR ones (Fig. 5F, G).

To further assess the spatial distribution, we applied a semiquantitative score to the immunohistochemistry sections by dividing the tumors in the rim and the core regions and by considering both the intensity and the area coverage of the staining. The immunostaining score was significantly higher in 4T1 tumors than in 67NR ones for LDHA, PDK1 and CAIX (Fig. 5H). Of note, only PDK1 showed a statistically significant marked difference staining between the rim and the core regions for the 4T1 tumors.

Taken together, these results suggest a relationship between marked tumor acidosis, enhanced glycolysis, and hypoxia with an invasive and aggressive phenotype.

## Discussion

Metabolic reprogramming is nowadays considered a hallmark of cancer and the presence of an acidic tumor microenvironment can select for the survival of cancer cells with adaptive advantages that lead to a more invasive and malignant phenotype [4, 42] in several solid tumors, including breast cancer. Despite the well-established role of tumor acidosis in promoting cancer aggressiveness and therapy resistance, there is an astonishing lack of in vivo imaging studies at the preclinical level and with potential clinical feasibility, that investigated this association. In this work, we selected two isogenic murine breast cancer models, 4T1 and 67NR, which display divergent metastatic potential [26], and characterized both the derived cell lines and orthotopic breast tumors for their metabolic properties, tumor acidosis, and vascularization via MRI-based techniques. We observed a different metabolic phenotype between 4T1 and 67NR both in cells and in the orthotopic primary tumors that contributed to distinct metastatic behaviors. We found that, in contrast to the 67NR tumors, the 4T1 tumors were not only more acidic, but also showed increased tumor pH_e_ heterogeneity. We demonstrated that highly metastatic tumors present a marked spatial pH_e_ heterogeneity, highlighting the strong relationship between tumor acidosis and the aggressive and metastatic potential.

Although several previous imaging-based studies have reported different metabolic and tumor microenvironment properties between these two cell lines, they are limited because of their ability to investigate the spatial heterogeneity within the whole tumor [7, 30, 43–46]. In fact, all these studies extracted information from only a small portion of the tumor (being the central part/central slice or the superficial region), lacking the full volume coverage of the tumor. Conversely, in this study, we accurately quantified the 3D distribution of the extracellular pH and other tumor features. Consistent with the well-established role of tumor heterogeneity in leading to long-term adverse outcomes, this improved in vivo spatial heterogeneity mapping of tumor acidosis enabled the robust differentiation of the two breast cancer lines with different malignant potentials.

Multiple studies have demonstrated that 4T1 tumor cells display increased glycolytic and oxidative phosphorylation activity. This metabolic flexibility allows them to adapt to different microenvironmental conditions, providing proliferative advantages and enhancing their metastatic potential [26, 28]. Given these findings, we sought to determine whether the acidosis observed in these tumors is associated with metabolic changes that contribute to their increased degree of malignancy. Although in this study we focused on an altered glycolytic metabolism, other metabolic pathways (glutaminolysis and lipid metabolism) can contribute to the observed acidity. Specifically, we aimed to explore the relationship between tumor acidosis and the metabolic adaptations that may drive the aggressive behavior of 4T1 cells. The upregulation of the LDH-A and PDK-1 genes has been associated with increased metastatic potential and their expression is essential for tumor growth and the establishment of malignant phenotype [47, 48]. Here, we showed that 4T1 cells presented increased LDH-A and PDK1 expression at both the protein and mRNA levels, thus exhibiting a more glycolytic phenotype associated with their aggressiveness and metastatic potential. Additionally, in 4T1 tumors, the immunohistochemical staining for LDH-A and PDK-1 showed marked expression within the whole tumor section, which correlated also with the stronger tumor acidosis observed with CEST pH imaging, whereas 67NR tumors presented less protein quantification for both markers. Recently, LAMP2 has been considered a valuable biomarker of tumor acidosis, and its overexpression has been correlated with breast cancer progression to malignant stages, as it allows the adaptation and survival of cancer cells in low-pH tumor microenvironments. For these reasons, the greater expression that we observed in 4T1 tumors through western blot, immunofluorescence, and immunohistochemistry, could be related to the greater degree of tumor acidosis observed with CEST-MRI.

The development of malignant and invasive breast cancer phenotypes is sustained by strongly acidic tumor cells that contribute to the creation of a heterogeneous tumor microenvironment, with local hypoxic and poorly vascularized tumor regions [49]. Furthermore, recent studies have demonstrated a strong correlation between metabolism and vascularity [50]. The DCE-MRI technique is widely used to evaluate tumor perfusion and vessel permeability, with promising results in monitoring vascular changes during breast cancer progression through malignant stages [51, 52]. In our study, we detected different vascular patterns between the two tumor models by both in vivo and ex vivo analyses. We observed a reduced vessel permeability in 4T1 tumors with a significant spatial heterogeneity, as reported by the parametric maps of K^trans^, with higher values in the rim and lower values in the inner tumor areas, because of the presence of necrotic regions. The quantification of CD31-positive endothelial cells in tumor specimens highlighted a greater number of vessels in 67NR tumors than in 4T1 tumors. Our results are in accordance with those of previous works on these two tumor types, which revealed greater perfusion and vascularity for the 67NR tumors than for 4T1 tumors, with different intratumoral regions of high and low perfusion that correlated with CD31 expression [44, 53].

The reduced perfusion and vascularity in 4T1 tumors, particularly in the inner tumor regions, is expected to be associated with intratumoral hypoxia, an important characteristic of highly malignant tumors [49]. Specifically, the upregulation of carbonic anhydrase IX (CAIX), which catalyzes the reverse hydration of CO_2_ to bicarbonate ions and protons and promotes the acid-base balance in cells, by hypoxia-inducible factor 1 has been positively correlated with malignant and glycolytic phenotypes [54]. The greater expression of CAIX observed in 4T1 tumors than in 67NR tumors is reflected in increased extracellular acidosis and higher levels of hypoxia. Furthermore, CAIX inhibition was previously shown to reduce in vivo tumor growth and metastasis formation, suggesting that CAIX is a poor prognostic biomarker for metastases [55]. The higher CAIX expression observed in 4T1 tumors is associated with a greater number and larger metastases observed in the lungs of 4T1 tumor-bearing mice, hence leading to a more invasive phenotype (Suppl. Figure 2). Consistently, we detected lower staining of the hypoxia reporter pimonidazole in 67NR tumors than in 4T1 ones which is in agreement with previous research [53]. While this study advances our understanding of the spatial heterogeneity of intratumoral acidosis in the context of metastatic breast cancer, we acknowledge several important limitations. First, the use of only two breast cancer cell lines restricts the generalizability of our findings, particularly in the context of inter-tumoral heterogeneity. Second, although we have previously characterized these models in terms of functional metabolism [29], the present study did not explore how tumor heterogeneity and pH intersect with nutrient-dependent metabolic adaptations. Moreover, while ¹⁴C-based approaches provide valuable information on pathway activity, ¹³C-labeled metabolic flux analysis and isotopologue distribution profiling would offer a more detailed and quantitative understanding of intracellular metabolic destiny. Finally, our analysis focused primarily on glucose metabolism, without assessing the contribution of other key nutrients such as glutamine and fatty acids, which are known to support cancer metabolism and may interact with microenvironmental conditions such as acidosis. Future studies incorporating a broader panel of cell lines, stable isotope tracing, and multi-nutrient metabolic profiling will be essential to fully elucidate the interplay between tumor heterogeneity, pH, and metabolic plasticity.

## Conclusions

Taken together, these findings show that a greater metabolic plasticity, coupled with a reduced perfusion and increased hypoxia, results in an acidic tumor microenvironment that is correlated with an aggressive and metastatic breast cancer phenotype. The CEST-pH imaging technique, which provides accurate measurements of tumor acidosis, was able to distinguish between malignant and non-malignant breast cancer phenotypes, supporting a strong correlation between tumor acidosis and aggressiveness. Additionally, the ability to investigate in vivo spatial tumor pH_e_ heterogeneity further improved the differentiation between more and less invasive breast cancer phenotypes.

## Electronic supplementary material

Below is the link to the electronic supplementary material.

**Supplementary Material 1**: **Supplementary Fig. 1.** Basal (A) and compensatory (B) glycolysis rates were extrapolated from the Glyco rate assay and normalized to the protein content. Data represent the means ± SEMs. *n* = 3 biological replicates in at least either duplicate or technical triplicate. Student’s t-test. Basal (C) and maximal (D) respiration rates were extrapolated from the Mito Stress Test and normalized to the protein content. Data represent the means ± SEMs. *n* = 3 biological replicates in at least either duplicate or technical triplicate. Student’s t-test. *** *P* < 0.001; **** *P* < 0.0001. **Supplementary Fig. 2.** 4T1 tumor-bearing mice developed a greater number of lung metastases. Representative H&E staining of lung metastases from 4T1 (top) and 67NR (bottom) tumor-bearing mice. Amplification x4. **Supplementary Fig. 3**. Correlation of the tumor acidity score with TGR.

## Data Availability

The datasets generated and/or analysed during the current study are available from the corresponding author on reasonable request.

## Abbreviations

CEST: Chemical exchange saturation transfer
CAIX: Carbonic anhydrase IX
DCE: Dynamic contrast enhanced
GlycoPER: Glycolytic proton efflux rate
LAMP2: Lysosomal-associated membrane protein 2
LDH-A: Lactate dehydrogenase A
MRI: Magnetic resonance imaging
MVD: Microvessel density
OCR: Oxygen consumption rate
OXPHOS: Oxidative phosphorylation
PDK1: Pyruvate dehydrogenase kinase 1
pH_e_: Extracellular pH
TGR: Tumor Growth Rate
TME: Tumor microenvironment
