# Supplementary material for: In vivo imaging of the spatial heterogeneity of intratumoral acidosis (pH) as a marker of the metastatic phenotype in breast cancer
Source: Breast Cancer Res. 2025 Jun 23;27:112. doi: 10.1186/s13058-025-02065-y (PMC12183868; doi:10.1186/s13058-025-02065-y)
Supplement: Supplementary file 1 — Supplementary Material 1: Supplementary Fig. 1. Basal (A) and compensatory (B) glycolysis rates were extrapolated from the Glyco rate assay and normalized to the protein content. Data represent the means ± SEMs. n = 3 biological replicates in at least either duplicate or technical triplicate. Student’s t-test. Basal (C) and maximal (D) respiration rates were extrapolated from the Mito Stress Test and normalized to the protein content. Data represent the means ± SEMs. n = 3 biological replicates in at least either duplicate or technical triplicate. Student’s t-test. *** P < 0.001; **** P < 0.0001. Supplementary Fig. 2. 4T1 tumor-bearing mice developed a greater number of lung metastases. Representative H&E staining of lung metastases from 4T1 (top) and 67NR (bottom) tumor-bearing mice. Amplification x4. Supplementary Fig. 3. Correlation of the tumor acidity score with TGR. [file 13058_2025_2065_MOESM1_ESM.docx]

**Supplementary Information**

***In vivo* imaging of the spatial heterogeneity of intratumoral acidosis (pH) as a marker of the metastatic phenotype in breast cancer**

Alessia Corrado^1^, Nicla Lorito^2^, Annasofia Anemone^3^, Antonella Carella^1^, Daisy Villano^3^, Elisa Pirotta^1^, Francesco Gammaraccio^3^, Angela Subbiani^2^, Marina Bacci^2^, Walter Dastrù^3^, Andrea Morandi^2^ and Dario Livio Longo^1^

^1^Institute of Biostructures and Bioimaging (IBB), National Research Council of Italy (CNR) – Via Nizza 52, 10126 Turin, Italy

^2^Department of Experimental and Clinical Biomedical Sciences, University of Florence – Viale Morgagni 50, 50134 Florence, Italy

^3^Department of Molecular Biotechnology and Health Sciences, University of Turin – Via Nizza 52, 10126 Turin, Italy


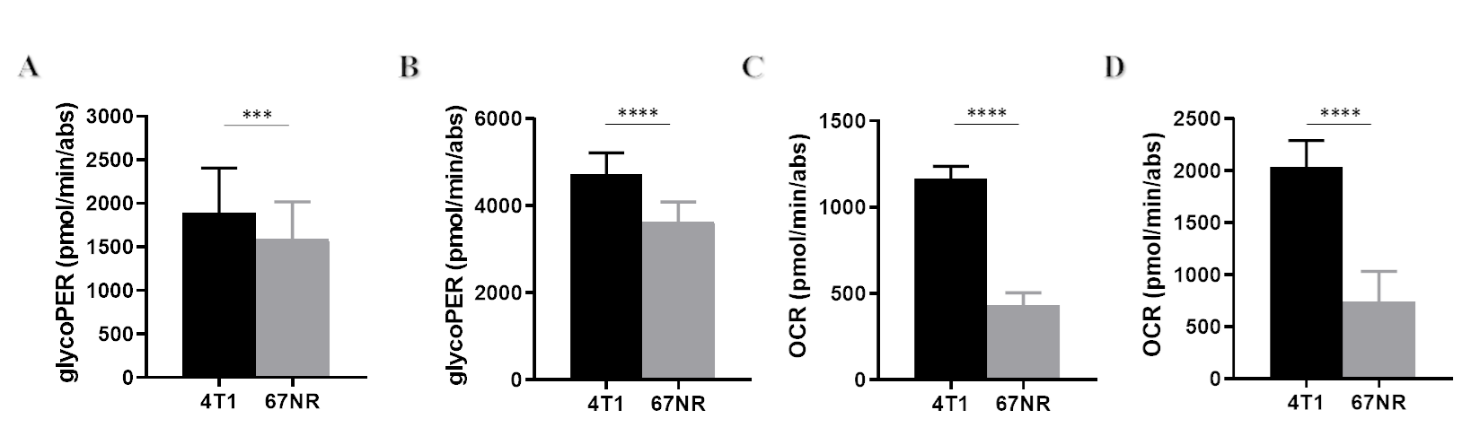


**Supplementary Figure 1**. Basal (A) and compensatory (B) glycolysis were extrapolated from Glyco Rate Assay, calculated as described in Methods, and normalized on protein content. Data represent means ± SEMs. n = 3 biological replicates in at least either duplicate or technical triplicate. Student’s t-test. Basal (C) and maximal (D) respiration were extrapolated from Mito Stress Test, calculated as described in Methods, and normalized on protein content. Data represent means ± SEMs. n = 3 biological replicates in at least either duplicate or technical triplicate. Student’s t-test. *** P<0.001; **** P<0.0001.


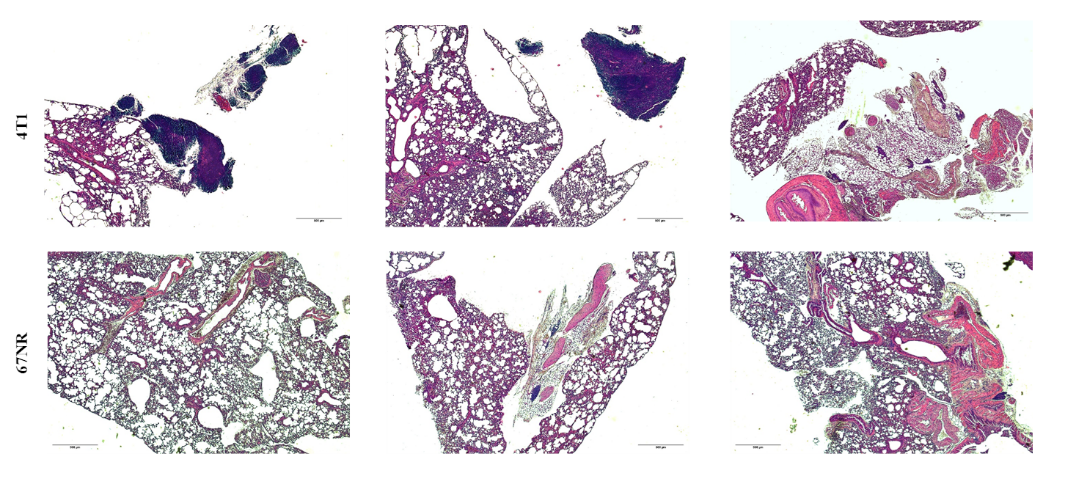


**Supplementary Figure 2**. 4T1 tumor-bearing mice developed a higher number of lung metastases. Representative H&E staining of lung metastasis of 4T1 (top) and 67NR (bottom) tumor-bearing mice. Amplification x4.

**Supplementary Figure 3.** Correlation between tumor growth rate and acidity score values
